# Development of *Metarhizium anisopliae *and *Beauveria bassiana *formulations for control of malaria mosquito larvae

**DOI:** 10.1186/1756-3305-4-23

**Published:** 2011-02-22

**Authors:** Tullu Bukhari, Willem Takken, Constantianus JM Koenraadt

**Affiliations:** 1 Laboratory of Entomology, Wageningen University, Wageningen, The Netherlands

## Abstract

**Background:**

The entomopathogenic fungi *Metarhizium anisopliae *and *Beauveria bassiana *have demonstrated effectiveness against anopheline larvae in the laboratory. However, utilising these fungi for the control of anopheline larvae under field conditions, relies on development of effective means of application as well as reducing their sensitivity to UV radiation, high temperatures and the inevitable contact with water. This study was conducted to develop formulations that facilitate the application of *Metarhizium anisopliae *and *Beauveria bassiana *spores for the control of anopheline larvae, and also improve their persistence under field conditions.

**Methods:**

Laboratory bioassays were conducted to test the ability of aqueous (0.1% Tween 80), dry (organic and inorganic) and oil (mineral and synthetic) formulations to facilitate the spread of fungal spores over the water surface and improve the efficacy of formulated spores against anopheline larvae as well as improve spore survival after application. Field bioassays were then carried out to test the efficacy of the most promising formulation under field conditions in western Kenya.

**Results:**

When formulated in a synthetic oil (ShellSol T), fungal spores of both *Metarhizium anisopliae *and *Beauveria bassiana *were easy to mix and apply to the water surface. This formulation was more effective against anopheline larvae than 0.1% Tween 80, dry powders or mineral oil formulations. ShellSol T also improved the persistence of fungal spores after application to the water. Under field conditions in Kenya, the percentage pupation of *An. gambiae *was significantly reduced by 39 - 50% by the ShellSol T-formulated *Metarhizium anisopliae *and *Beauveria bassiana *spores as compared to the effects of the application of unformulated spores.

**Conclusions:**

ShellSol T is an effective carrier for fungal spores when targeting anopheline larvae under both laboratory and field conditions. Entomopathogenic fungi formulated with a suitable carrier are a promising tool for control of larval populations of malaria mosquitoes. Additional studies are required to identify the best delivery method (where, when and how) to make use of the entomopathogenic potential of these fungi against anopheline larvae.

## Background

Recently, theoretical and experimental studies have shown the potential of entomopathogenic fungi as next generation agents for the control of malaria mosquitoes [1-5] However, most of this work has focused on targeting adult mosquitoes. Larval control has a convincing history of malaria eradication and recent studies have also shown this approach to be highly effective [6-11]. It is, therefore, worthwhile to investigate the ability of entomopathogenic fungi to control mosquito larvae and the feasibility of their operational use.

Our previous work showed the efficacy of *Metarhizium anisopliae *(ICIPE-30) and *Beauveria bassiana *(IMI- 391510) spores in infecting and killing larvae of *Anopheles stephensi *and *An. gambiae *under laboratory conditions [12]. Other isolates of *M. anisopliae *and *B. bassiana *have also been shown to affect culicine and anopheline larvae [13-17]. The main infection sites were the feeding and respiratory apparatus [16]. Most of these studies had been carried out in the laboratory and proved the application of dry fungal spores to be more effective than the application of formulated spores [13,14,18]. Applying dry spores in the field, however, has certain limitations. Fungal spores are hydrophobic by nature so when applied in an aquatic environment, they clump together, reducing the area that is effectively covered. As a result, massive amounts of fungal spores are required. Contact with water also disrupts the infection process. Attachment of spores to the host is an important step of the infection process. The outer layer of spores has highly organised surface proteins known as rodlets, which are mainly responsible for attachment to the host [19]. For successful infection, germination should follow spore attachment to the host. When dry fungal spores are applied to an aquatic habitat, typical for mosquito larvae, the nutrients in the water are usually sufficient to stimulate germination in the spores following water intake [20,21]. Once a spore germinates, the outer layer is ruptured reducing the chance of attachment to the host. Water contact, thus, reduces the pathogenicity of the floating spores. In addition, dry unformulated fungal spores are more exposed to UV radiation and high temperatures, which are known to negatively affect spore persistence and germination rate [22,23].

In addition to strain selection and genetic modification, formulation can have a considerable impact on improving the efficacy of biopesticides. An ideal formulation aids the handling and application of the biopesticides, as well as increases its efficacy by improving contact with the host and protecting the active agent from environmental factors [24]. Considering the surface feeding behaviour of anopheline larvae, any formulation intended to infect them should spread the fungal spores over the water surface [25,26]. The larvae are then most likely to come in contact with spores. The spores should spread uniformly, providing equal coverage, over the entire treated area. In addition, spores should be prevented from germinating before host attachment, and at least to some extent be protected from environmental factors. In this context we developed and tested dry (organic and inorganic), oil (mineral and synthetic) and water-based formulations of *M. anisopliae *and *B. bassiana *for their efficacy against anopheline larvae.

The objectives of this study were to (a) develop formulations suitable for the positioning (water surface or bottom) and uniform spread of *M. anisopliae *and *B. bassiana *spores, (b) assess the efficacy of selected spore formulations in killing anopheline larvae, (c) assess the selected formulations for their potential to increase spore persistence, and (d) assess the potential of formulations to suppress populations of mosquito larvae in a field situation.

## Methods

### Mosquitoes

*Anopheles gambiae s.s. *(Suakoko strain, courtesy of Prof. M. Coluzzi, reared in laboratory for 23 years) and *An. stephensi *(Strain STE 2, MR4 no. 128, origin India, reared in laboratory for 2 years after obtaining the eggs from MR4) were reared separately, but under similar conditions, in climate-controlled rooms at Wageningen University, The Netherlands. The temperature was maintained at 27 ± 1°C. Relative humidity was set at 70 ± 5% and the rooms had a 12L:12D photoperiod. Larvae were kept in plastic trays filled with tap water. First instar larvae were fed on Liquifry No. 1 (Interpet Ltd., Surrey, UK) while older instar stages were fed on Tetramin^® ^(Tetra, Melle, Germany). The resulting pupae were transferred to holding cages (30 × 30 × 30 cm) in small cups, where they emerged as adults with *ad libitum *access to 6% glucose water. The female mosquitoes were blood-fed with the Hemotek membrane feeding system. Human blood (Sanquin^®^, Nijmegen, The Netherlands) was used for this purpose and mosquitoes could feed on it through a Parafilm M^® ^membrane. Eggs were laid on moist filter paper, and were subsequently transferred to the larval trays. For the field bioassays *An. gambiae *s.s. eggs (Kisumu, strain, reared in laboratory for 8 years) were obtained from the Kenya Medical Research Institute (KEMRI) and reared at the Ahero Multipurpose Development Training Institute (AMDTI), Kenya. Rearing was carried out under local climate conditions (described below) and larvae were fed on Tetramin^®^.

### Fungal spores

*Metarhizium anisopliae *(ICIPE-30) and *Beauveria bassiana *(IMI- 391510) spores were obtained from the Department of Bioprocess Engineering, Wageningen University, and stored as dry spores in Falcon™ tubes at 4°C. *Metarhizium anisopliae *spores are olivaceous green, cylindrical and 2.5-3.5 μm long while *B. bassiana *spores are hyaline, spherical or sub-spherical and have a diameter of 2-3 μm [27].

### Carrier materials

Wheat flour, white pepper, WaterSavr (WaterSavr™, Sodium bicarbonate version, Flexible Solutions International Ltd., Victoria BC, Canada), 0.1% Tween 80 aqueous solution, Ondina oil 917 (Shell Ondina^® ^Oil 917, Shell, The Netherlands) and ShellSol T (Shellsol T^®^, Shell, The Netherlands) were tested for their potential as carrier of fungal spores. Wheat flour and white pepper served as organic dry carriers. These were tested because anopheline larvae are known to aggregate around and feed on powdered organic materials (wheat flour, alfalfa flour, blood meal and liver powder) even when a choice of inorganic materials (chalk, charcoal and kaolin) is also available [28]. One inorganic dry powder, known as WaterSavr, was also tested. WaterSavr consists of fine bicarbonate granules that self-spread over the water surface forming a thin layer which has been shown to reduce evaporation [29]. Its biodegradability, safety and surface-spreading features made it a suitable candidate for inclusion in our tests. Surfactants, such as Tween 80, can be used to overcome the hydrophobic nature of fungal spores and form a homogeneous aqueous solution. Fungal spores formulated in Tween 80 have been used in bioassays to test the efficacy of fungal spores against mosquito larvae [13,16,30-34]. ShellSol T is a synthetic isoparaffinic hydrocarbon solvent. Ondina oil 917, slightly denser than ShellSol T, is a highly refined mineral oil. Both ShellSol T and Ondina oil 917 have been successfully used as carrier for fungal spores to target the adult stage of mosquitoes [1,35].

### Formulations

The first selection of carriers suitable for formulating entomopathogenic fungal spores consisted of a test in which the carrier material was evaluated for its ability to spread over the water surface. For this purpose, plastic trays (25 × 25 × 8 cm) were filled with 1 L of tap water and the carriers applied on the water surface (441 cm^2^). The least amount of each carrier required to cover the entire surface was recorded. Once that amount was determined, *M. anisopliae *spores (10 mg, ~ 4.7 × 10^8 ^spores) were added to the carriers. The quantity of the carriers was increased to make a consistent suspension or mixture of fungal spores and carriers. The resulting formulations were applied to select the carriers that spread the spores evenly over the water surface evenly. *Metarhizium anisopliae *spores were used because of their colour (olivaceous green) which made it easy to visualize them whilst spreading.

### Efficacy of formulations against *Anopheles gambiae *larvae

The next step consisted of testing selected formulations against *An. gambiae *larvae in laboratory bioassays. Bioassays were performed under climatic conditions similar to the mosquito rearing. Plastic trays (25 × 25 × 8 cm) were filled with 1 L of tap water and allowed to acclimatise overnight. Fifty second-instar larvae were added to each tray. Unformulated or formulated spores were applied to the water surface of each tray. The number of larvae that died or pupated was recorded daily for the next eight days. For each treatment, the carrier alone (in the same quantity as in the formulation) served as the control. In the case of unformulated spores, the control was untreated tap water. The larvae were provided with Tetramin^® ^as food at the rate of 0.2 - 0.3 mg/larva per day. The experiments were replicated three times.

### Pathogenicity of floating unformulated spores over time

A third experiment was performed to evaluate how the pathogenicity of fungal spores is affected by being in contact with water over a time period of seven days. At the start, 15 plastic trays (same size as above) were each filled with one liter of water. These trays were kept overnight in a climate-controlled room to acclimitise. *Metarhizium anisopliae *spores were applied to the water surface in five trays (10 mg per tray). Similarly, 10 mg of *B. bassiana *spores (~ 2 × 10^9 ^spores) were applied on the water surface in five other trays. The remaining five trays served as the control. After one day, 50 second-instar *An. stephensi *larvae were added to one of the trays treated with *M. anisopliae *spores, *B. bassiana *spores and one untreated control tray. Similarly larvae were added to remaining trays after either 2, 3, 5 or 7 days after fungal treatment. The mortality and/or pupation was followed for 9 days. The larvae were fed at the same rate as mentioned before. This experiment was replicated three times.

### Effect of formulation on persistence of pathogenicity

Based on the results of the formulation experiments, the carriers WaterSavr and ShellSol T were selected and tested further for their ability to increase the persistence of pathogenicity in fungal spores in contact with water. Unformulated and formulated (either with WaterSavr or ShellSol T) *M. anisopliae *and *B. bassiana *spores were applied to plastic trays containing 1 L of acclimatized water. One replicate consisted of 18 trays. A pair of trays was applied with one of the following nine treatments: (1) 10 mg of dry *M. anisopliae *spores, (2) 10 mg of dry *B. bassiana *spores, (3) *M. anisopliae *spores mixed with WaterSavr (10 mg/130 mg), (4) *B. bassiana *spores mixed with WaterSavr (10 mg/130 mg), (5) *M. anisopliae *spores mixed with ShellSol T (10 mg/200 μl), (6) *B. bassiana *spores mixed with ShellSol T (10 mg/200 μl), (7) WaterSavr (130 mg) only, (8) ShellSol (200 μl) only or (9) no treatment. Trays treated with WaterSavr or ShellSol T without fungal spores and the untreated trays served as control for their respective treatments. Fifty second-instar *An. stephensi *larvae were added to one tray of each pair on the same day the fungal spores were applied. The same number of larvae was added to the other tray of the pair on the seventh day (based on the results of the previous experiment). The larvae were checked for mortality or pupation for the following 10 days after being added to the trays. The experiment was replicated three times. The trays were topped up with acclimatised tap water, every other day, to compensate for evaporation.

### Field bioassays

To evaluate the efficacy of unformulated and formulated fungal spores in the field, experiments were carried out in Kenya in May and June, 2010. The experiments were conducted in a restricted part of the Ahero Multipurpose Development and Training Institute (AMDTI) compound. This institute is located 24 km southeast of Kisumu, in western Kenya (0°10'S, 34°55'E). Malaria is highly endemic in this region and transmission occurs throughout the year. A mean annual *Plasmodium falciparum *sporozoite inoculation rates (EIR) of 0.4-17 infective bites per year has been shown by recent studies for this region [36]. The region has an annual mean temperature range of 17°C to 32°C, average annual rainfall of 1,000 - 1,800 mm and average relative humidity of 65% [37].

**Figure 1:**
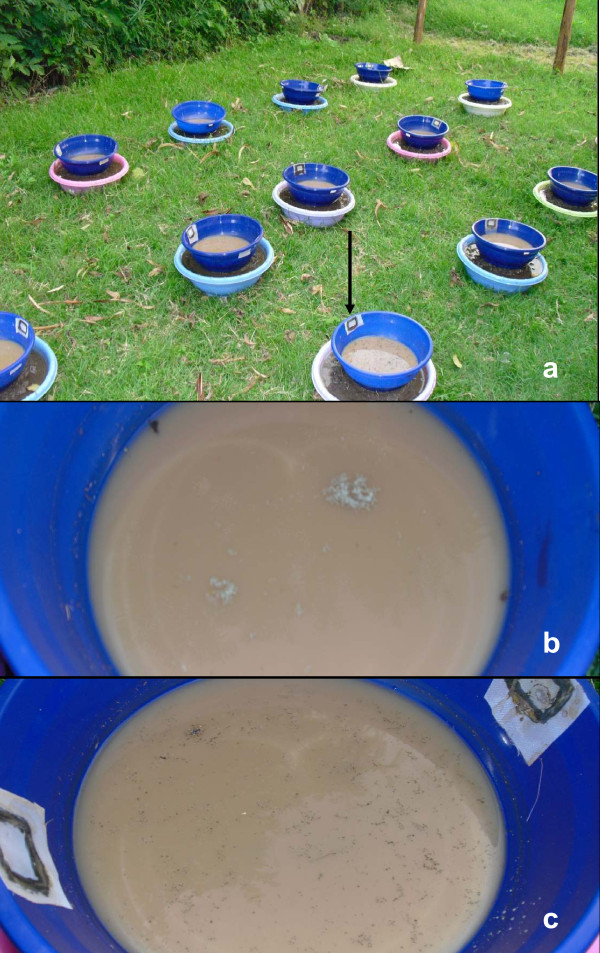
**Field bioassays**. **(a) **Forty *An. gambiae *larvae were placed in plastic containers (with nylon screened holes, indicated by an arrow) with a soil layer (2 cm) at the bottom and a 3 cm layer of water. The screened holes were a precautionary measure to retain larvae in the tubs in case of overflow due to heavy rain. The plastic containers were placed in larger tubs, also filled with soil and water, to prevent ants from access to the bioassays. **(b) **Unformulated (dry) *Metarhizium anisopliae *(10 mg) spores applied on the water surface. Note the two large clumps just outside the centre of the containers. **(c) **Shellsol T-formulated *Metarhizium anisopliae *(10 mg) spores applied on the water surface. Note that spores are spread more evenly over the surface by ShellSol T than dry spores (Figure b).

Bioassays were conducted outdoors in 33 plastic containers (0.30 m diameter). The plastic containers had two nylon-screened holes (3 cm^2^), close to the brim, allowing excess rain water to flow out while retaining the larvae. Dry soil from a rice paddy at the Ahero irrigation scheme (4 km from AMDTI) was softened up by adding water. The softened soil was placed at the bottom of each plastic container to form a 2 cm thick layer. One L of tap water was then added to each plastic container. The water level was 3 cm above soil level and exposed a surface area of 450 cm^2^. Each plastic container was placed in a larger tub that also had a bottom layer of soil but was filled with water to the top. The larger tubs were employed to prevent ants from accessing the plastic container inside. Forty second-instar *An. gambiae s.s. *larvae, were added to each container. The large tubs, with the containers inside, were arranged in three rows 0.5 m apart from each other (Figure 1a).

Dry and ShellSol T formulated spores of both fungal species were tested. ShellSol T was the only formulation that successfully met the criteria investigated in the laboratory studies. Two different concentrations (10 mg spores/200 μl ShellSol T and 20 mg spores/230 μl ShellSol T) of both *M. anisopliae *and *B. bassiana *spores were tested. For the larger amount of spores, 230 μl ShellSol T was required to make a consistent suspension. Each treatment was randomly applied to three plastic containers. The 11 treatments consisted of dry *M. anisopliae *spores (10 mg and 20 mg), dry *B. bassiana *spores (10 mg and 20 mg), ShellSol T formulated *M. anisopliae *spores (10 mg/200 μl and 20 mg/230 μl), ShellSol T formulated *B. bassiana *spores (10 mg/200 μl and 20 mg/230 μl) and only ShellSol T (200 μl and 230 μl) while the one remaining tub was untreated. The ShellSol T (200 μl and 230 μl) and the untreated container served as control for their respective treatments. The number of larvae that died in the containers could not be recorded because it was difficult to recover them in the turbid water and/or bottom soil. Therefore, larval survival was assessed as the number of pupae produced. No food was provided to the larvae after being placed in the container. The plastic containers were checked twice daily (for the following 15 days) and pupae were removed with a dipper. To prevent oviposition or emergence of local mosquitoes in the water of larger tubs in which treated plastic containers were placed, Aquatain (a silicone-based oil) was applied to the water surface [38]. Water (0.5 L, kept outdoors in Jerry cans) was added to every plastic container when the water level had been reduced by evaporation to less than 1 cm. Meteorological data was obtained from the National Irrigation Board (NIB) research station located approximately 4 km from the experimental site. Water surface (5 mm top layer) temperature was measured daily at the same time, in each container, with a digital thermometer (GTH 175/Pt, Greisinger electronics, Germany).

### Statistical analysis

Differences in larval survival were analysed using Cox regression [39]. The survival of larvae treated with formulated or unformulated fungal spores were compared with their respective control larvae and the resulting Hazard Ratio (HR) values were used to evaluate differences in mortality rates. The proportional hazard assumption of Cox regression was tested by plotting the cumulative hazard rates against time for the treated and control groups to confirm that the resulting curves did not cross [40].

To test the pathogenicity of fungal spores over time, HR's were computed for larvae exposed to spores floating on water for different time periods. In addition, the arcsine-square root transformed proportions of dead larvae were compared directly, after being corrected for their respective controls using the Abbott's formula, by a one-way ANOVA and LSD post-hoc test of the arcsine transformed mortality proportion [41]. Similarly, the persistence of pathogenicity in formulated and unformulated spores was also compared. The arcsine-square root transformed proportions of larvae that pupated in the field trial were compared by one-way ANOVA and LSD post-hoc tests. All the analyses were performed using SPSS version 15 software (SPSS Inc. Chicago, IL, USA).

## Results

### Formulations

In the case of both ShellSol T and Ondina oil 917, 100 μl of the oil was required to cover a water surface of 441 cm^2^. The amounts could not be determined for 0.1% Tween 80 and wheat flour. Tween 80 solution could not be visualised as it is colourless. The wheat flour formed clumps rather than spreading. White pepper spread across the water surface evenly and 30 mg of it was sufficient to cover the entire surface area. Similarly 130 mg of Watersavr spread and covered the water surface of 441 cm^2 ^(Table 1). After determining these amounts, 10 mg of *Metarhizium anisopliae *spores was added to each of the carriers. The quantity of ShellSol T and Ondina oil 917 had to be doubled (200 μl) to form a homogenous suspension. In case of the 0.1% Tween 80 solution, 4 ml was required to form a consistent suspension. Wheat flour was not tested further because of clumping. The quantity of white pepper and WaterSavr (30 mg and 130 mg respectively) required for covering the water surface (441 cm^2^) was also enough to form a consistent mixture with 10 mg of fungal spores (Table 1). Formulations, apart from the 0.1% Tween 80 solution which caused the spores to sink, resulted in a fairly uniform spread of fungal spores on the water surface (Table 1). Therefore 0.1% Tween 80 solution was not tested further.

**Table 1:** Carriers tested for their ability to spread spores and the composition of formulations tested

	Amount required to			
			
Carrier	cover 441 cm^2^	mix 10 mg of fungal spores	Spore spreading	Composition of formulations tested in bioassays (fungal spores/carrier)
Wheat flour	--	--	--	--
0.1% Tween 80	--	4 ml	causes spores to sink	--
White pepper	30 mg	30 mg	on the water surface	10 mg/30 mg
WaterSavr	130 mg	130 mg	on the water surface	10 mg/130 mg
Ondina oil 917	100 μl	200 μl	on the water surface	10 mg/200 μl
ShellSol T	100 μl	200 μl	on the water surface	10 mg/200 μl

The amount of each carrier required to cover a water surface area of 441 cm^2^, the amount required to form a consistent mixture with 10 mg of *Metarhizium anisopliae *spores, the ability of the carriers to spread the spores over the water surface and the composition of formulations with suitable carriers

-- Not Tested or could not be determined

### Efficacy of formulations against *Anopheles gambiae *larvae

**Figure 2:**
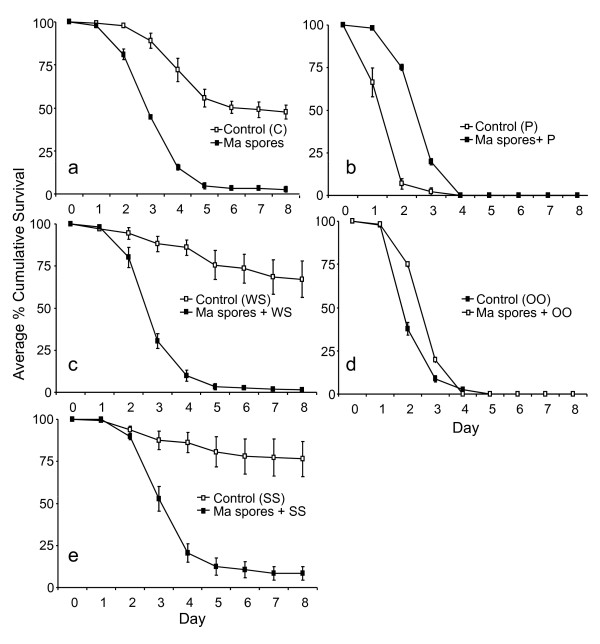
**Laboratory bioassays to test the efficacy of unformulated and formulated *Metarhizium anisopliae *spores**. The average percentage cumulative survival (±S.E.) of *An. gambiae *larvae (n = 3) exposed to **(a) **Unformulated *Metarhizium anisopliae *spores (control (C) and unformulated spores (Ma spores) **(b) **Pepper (control (P)) and Pepper formulated spores (Ma spores + P) **(c) **WaterSavr (control (WS)) and WaterSavr formulated spores (Ma spores + WS) **(d) **Ondina Oil (control (OO)) and Ondina oil formulated spores (Ma spores + OO) **(e) **ShellSol T (control (SS)) and ShellSol T formulated spores (Ma spores + SS) over 8 days post-treatment. Larvae that pupated are included as surviving.

Bioassays were conducted with unformulated *M. anisopliae *spores (10 mg) and *M. anisopliae *spores formulated in pepper (10 mg/30 mg), WaterSavr (10 mg/130 mg), ShellSol T (10 mg/200 μl )) or Ondina oil 917 (10 mg/200 μl) against *An. gambiae *larvae. Only 2.7 ± 1.8% of the larvae treated with unformulated *M. anisopliae *spores pupated while 47.6 ± 3.9% pupated in the relevant control. The treated larvae had a nearly two times higher daily risk of mortality as compared to the untreated control larvae (HR (95%CI) = 1.8 (1.4-2.4), Table 2, Figure 2a). WaterSavr formulation reduced the pupation of the larvae from 67.2 ± 10.6% to 1.3 ± 0.6%, exposing the formulation-treated larvae to nearly three times higher daily risk of mortality as compared to the control (Table 2, Figure 2c). With the ShellSol T formulation 1.3 ± 0.6% of the treated larvae pupated while the larvae treated with ShellSol T (without fungal spores) showed 85.4 ± 14.5% pupation. Larvae exposed to ShellSol T formulated spores of *M. anisopliae *had a mortality risk four times higher compared to larvae treated with ShellSol T only (HR (95%CI) = 3.7 (2.5-5.4), Table 2, Figure 2e). However, with white pepper and Ondina oil there was no significant difference in the mortality of larvae treated with the formulation or the carrier alone, or the formulations and fungal spores together. Both pepper and Ondina oil 917 killed 100% larvae even without fungal spores (Table 2, Figure 2b and 2d). These two carriers were not tested further as the objective was to develop a formulation that enhances the spreading and efficacy of the fungal spores to infect and kill larvae.

**Table 2:** Percentage pupation and Hazard ratios of larvae exposed to tested formulations

	**Average % Pupation ± S.E**.			
			
Formulation	Control	Treatment	HR(95%CI)	p value
Unformulated	47.6 ± 3.9	2.7 ± 1.8	1.8 (1.4-2.4)	<0.001
White pepper	0	0	0.9 (0.7-1.2)	0.959
WaterSavr	67.2 ± 10.6	1.3 ± 0.6	2.7 (1.9-3.8)	<0.001
Ondina oil 917	0	0	1.0 (0.8-1.2)	0.806
ShellSol T	85.4 ± 14.5	1.3 ± 0.6	3.7 (2.5-5.4)	<0.001

Average percentage pupation (±S.E.) of *An. gambiae *larvae exposed to unformulated spores and formulated *Metarhizium anisopliae *spores (n = 3). The carrier in each formulation (White pepper, WaterSavr, Ondina oil 917 or ShellSol T) served as the control. In case of unformulated spores the control was completely untreated. Carrier and *Metarhizium anisopliae *spores together formed the treatment. Hazard ratio's (HR) indicate the mortality risk in the treatments as compared to their respective controls

### Pathogenicity of floating unformulated spores over time

**Figure 3:**
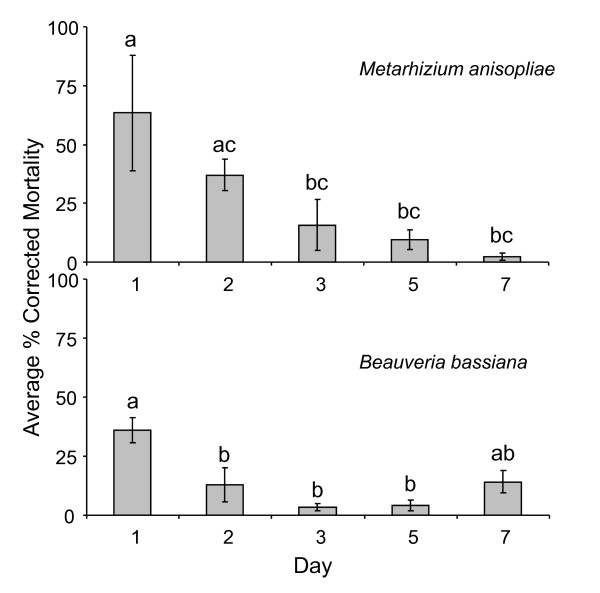
**Laboratory bioassays to test the persistence of floating unformulated fungal spores**. The average percentage corrected mortality (±S.E.) of *An. stephensi *larvae (n = 3) exposed to spores of *Metarhizium anisopliae *and *Beauveria bassiana *that had been floating on the water surface for 1, 2, 3, 5 or 7 days. Bars with letter in common show no significant difference (LSD post hoc test, α = 0.05).

The pathogenicity of dry *M. anisopliae *and *B. bassiana *spores was substantially reduced over a period of five days (Figure 3). *Anopheles stephensi *larvae exposed to *M. anisopliae *spores, applied to water seven days earlier, showed a similar pupation proportion as their control (Table 3). *Beauveria bassiana *spores lost their effectiveness after being in contact with water for three days. *Metarhizium anisopliae *spores lost their effectiveness after five days (Table 3). After seven days the control mortality was significantly higher than the mortality of larvae exposed to *M. anisopliae *treatment.

**Table 3:** Percentage pupation and Hazard ratio's of larvae exposed to unformulated floating fungal spores

	Average % Pupation (S.E.)			HR(95% CI) p value			
	
Treatment	Control	*Metarhizium anisopliae*	*Beauveria bassiana*	*Metarhizium anisopliae*		*Beauveria bassiana*	
Day 1	82.2 ± 13.5	36.1 ± 22.2	54.5 ± 8.4	5.2(3.4-8.0)	<0.001	3.2(2.0-5.0)	<0.001
Day 2	74.6 ± 10.8	46.7 ± 4.9	64.5 ± 5.3	2.5(1.7-3.6)	<0.001	2.0(1.3-3.0)	0.001
Day 3	96.0 ± 2.3	80.7 ± 8.5	92.8 ± 3.4	6.6(2.5-17.2)	<0.001	2.1(0.7-6.0)	0.169
Day 5	96.7 ± 1.7	87.4 ± 2.7	94.0 ± 3.0	4.4(1.6-11.8)	0.003	1.7(0.5-5.0)	0.347
Day 7	84.6 ± 2.6	84.7 ± 3.3	72.7 ± 2.9	0.3(0.2-0.6)	<0.001^a^	1.1(0.7-1.9)	0.625

Average percentage pupation (±S.E.) in the control and treated *An. stephensi *larvae exposed to *Metarhizium anisopliae *and *Beauveria bassiana *spores floating on the water surface for 1, 2, 3, 5 and 7 days (n = 3). The controls consisted of untreated trays filled with water at the same time as the treated trays. Hazard ratio's (HR) indicate the mortality risk of larvae as compared to the controls for both *Metarhizium anisopliae *and *Beauveria bassiana *spores

a. HR lower than 1 represents higher mortality in the control group.

### Effect of formulation on persistence of pathogenicity

**Figure 4:**
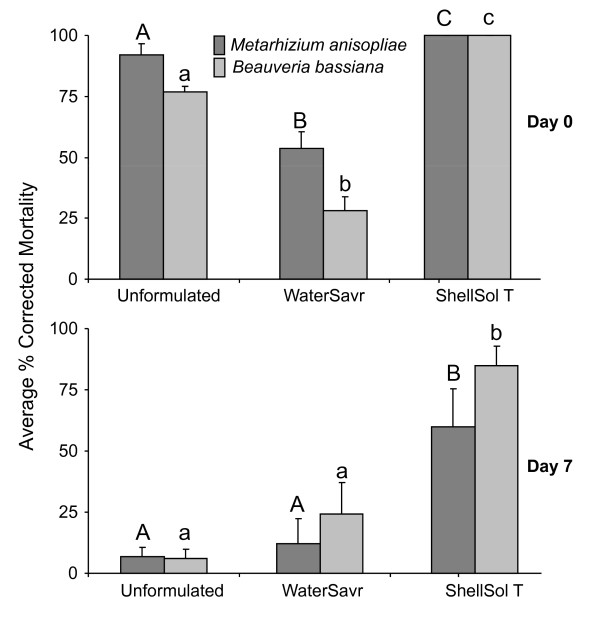
**Laboratory bioassays to test the persistence of formulated fungal spores**. The average percentage corrected mortality (±S.E.) of *An. stephensi *larvae (n = 3) exposed to unformulated, WaterSavr-formulated and ShellSol T-formulated *Metarhizium anisopliae *(Ma) and *Beauveria bassiana *(Bb) spores, immediately (Day 0) or seven days (Day 7) after application. Letters in common (upper case for Ma and lower case for Bb) show no significant difference (LSD post hoc test, α = 0.05).

Fungal spores formulated with ShellSol T were more persistent compared to the unformulated spores or spores formulated in WaterSavr. Seven days after application only ShellSol T formulated fungal spores (both *M. anisopliae *and *B. bassiana*) still caused significant mortality in the *An. stephensi *larvae (Table 4). Formulation in WaterSavr seemed to reduce the efficacy of fungal spores. When the *An. stephensi *larvae were exposed to WaterSavr-formulated *M. anisopliae *and *B. bassiana *spores, on the same day the fungal spores were applied, the corrected proportion larval-mortality was significantly lower as compared to larvae exposed to unformulated *M. anisopliae *and *B. bassiana *spores. Larvae exposed to *M. anisopliae *spores formulated with WaterSavr, applied that same day, had a lower mortality risk (HR (95% CI), 8.9 (4.4-18.1)) than those exposed to the unformulated spores (HR (95% CI), 44.6 (10.9-181.7)). There was no significant difference in the corrected proportion mortality of larvae exposed to unformulated and WaterSavr-formulated *M. anisopliae *spores, seven days after their application on water (Figure 4). Similar results were observed for *B. bassiana *spores. There was no significant difference between the corrected larval-mortality proportion due to unformulated and WaterSavr formulated *B. bassiana *spores, applied on water seven days before exposing the larvae. Also, the proportion larval mortality caused by WaterSavr-formulated *B. bassiana *spores was significantly lower than with ShellSol T-formulated *B. bassiana *spores (Figure 4).

**Table 4:** Hazard ratios of larvae exposed to (un)formulated fungal spores, 0 and 7 days post-application

Fungus	Day	Formulation	HR (95%CI)	p value
*Metarhizium anisopliae*	0	Unformulated	44.6 (10.9-181.7)	<0.001
		WaterSavr	8.9 (4.4-18.1)	<0.001
		ShellSol T	140.1 (18.4-1067.2)	<0.001
	7	Unformulated	1.0 (0.5-2.0)	0.816
		WaterSavr	1.1 (0.7-1.8)	0.477
		ShellSol T	1.5 (1.0-2.2)	0.030
*Beauveria bassiana*	0	Unformulated	36.1 (8.9-146.8)	<0.001
		WaterSavr	10.5 (4.7-23.5)	<0.001
		ShellSol T	137.9 (18.0-1053.4)	<0.001
	7	Unformulated	0.9 (0.4-1.7)	0.716
		WaterSavr	1.5 (0.1-2.3)	0.091
		ShellSol T	1.9 (1.3-2.9)	0.001

Hazard ratio's (HR) indicate the mortality risk of *An. stephensi *larvae exposed to unformulated, WaterSavr-formulated and ShellSol T-formulated *Metarhizium anisopliae *and *Beauveria bassiana *spores, 0 and 7 days after application (n = 3)

### Field bioassays

During the experimental period (15 days), the mean minimum and maximum temperatures were 15.7°C and 30.9°C, respectively, with a mean relative humidity of 54% and total rainfall of 19.4 mm. Water surface temperature ranged from 21°C to 38.8°C. Similar to the laboratory observations, unformulated spores clumped together on the water surface (Figure 1b) while ShellSol T-formulated fungal spores were uniformly spread (Figure 1c).

**Figure 5:**
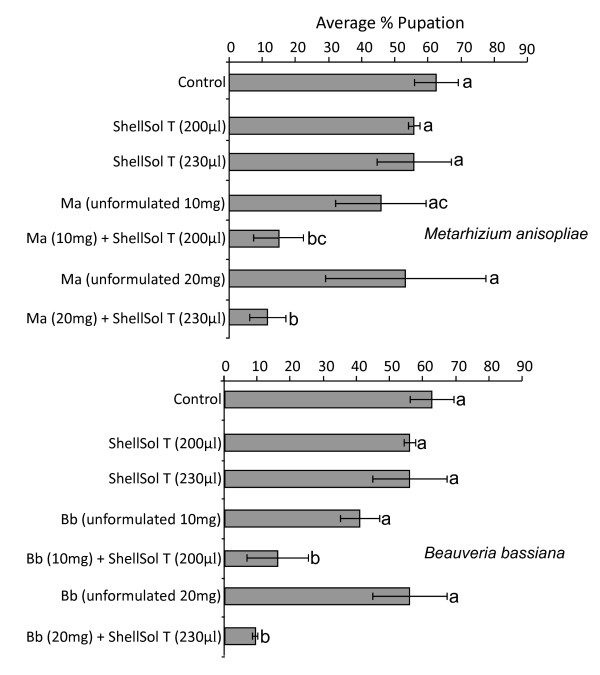
**Field bioassays testing the efficacy of fungal spores formulated in ShellSol T**. The average percentage pupation of *An. gambiae *larvae (n = 3) exposed to unformulated and ShellSol T formulated *Metarhizium anisopliae *(Ma) or *Beauveria bassiana *(Bb) spores at two doses, 10 mg/200 μl and 20 mg/230 μl. Controls included no treatment at all or treatment with only ShellSol T (200 μl or 230 μl). Letters in common show no significant difference (LSD post hoc test, α = 0.05).

The efficacy of unformulated fungal spores was found to be low under field conditions as compared to laboratory conditions. At dose rates of both 10 mg and 20 mg, the same (p > 0.05) level of pupation was observed in the *An. gambiae *larvae treated with unformulated *M. anisopliae *and *B. bassiana *spores as in the untreated *An. gambiae *larvae (Figure 5). As observed in the laboratory bioassays, ShellSol T on its own had no harmful effect on larval development and pupation. A similar proportion (p > 0.05) of larvae pupated in the containers treated with ShellSol T (200 μl and 230 μl) and the untreated containers (Figure 5).

The percentage pupation observed in *An. gambiae *larvae treated with ShellSol T-formulated *M. anisopliae *spores was 43% (low dose, 10 mg) and 49% (high dose, 20 mg) lower than that of the corresponding unformulated treatments. However for the lower dose (10 mg) the proportion of larvae that pupated was not significantly different (p = 0.08, Figure 5).

The percentage pupation observed in *An. gambiae *larvae treated with ShellSol T-formulated *B. bassiana *spores was 39% (low dose, 10 mg) and 50% (high dose, 20 mg) lower than that in the corresponding unformulated treatments. At both lower and higher dose the proportion of larvae that pupated was significantly different (p < 0.05, Figure 5).

## Discussion

The results of this study show how certain formulations can improve the ability of entomopathogenic fungus spores to spread over a water surface as well as increase their persistence. The results also show that better spreading and persistence leads to an enhanced efficacy of fungal spores. The study also demonstrates that both *M. anisopliae *and *B. bassiana *caused a high impact on the survival of *An. gambiae *s.s. larvae under field conditions, when formulated in Shellsol T.

*Anopheles stephensi *and *An. gambiae *larvae were found to be equally susceptible to unformulated *M. anisopliae *and *B. bassiana *spores [12]. This suggests that these fungi are likely to also affect other anopheline vector species.

Formulating fungal spores with Tween 80 and wheat flour was found to be unsuitable. Spores formulated with Tween 80 did not spread over the water surface, the primary feeding site of anopheline larvae, but sunk to the bottom [25,28]. Surfactants are known to impair attachment of the spore to the host so even if the spores were spread on the water surface they would not have been effective against anopheline larvae [20,42]. Wheat flour, although due to its organic nature could have served as a bait, did not spread the fungal spores over the water surface [28]. The wheat flour clumped together and sunk.

Powdered pepper and Ondina oil caused 100% mortality in anopheline larvae even without the fungal spores. Extracts of fruits of the *Piperaceae *family have been shown to be toxic for *Aedes aegypti *L. larvae [43], but the exact toxicity mechanism remains unclear. Although fungal spores were effectively spread with white pepper, pepper was considered an unsuitable carrier due to its own toxic effect on the anopheline larvae. Ondina oil, in the amount tested (200 μl), formed an oily layer over the water surface causing the larvae to suffocate. As compared to ShellSol T, Ondina oil is denser and evaporates less. This may explain the difference in the mortality observed with Ondina oil and ShellSol T controls. The amount of Ondina oil tested could not be reduced as, in that case, it was not possible to make a homogeneous suspension with the fungal spores.

Dry unformulated *M. anisopliae *and *B. bassiana *spores lost their pathogenicity five days after being applied to the water surface as the survival of larvae exposed to the fungal spores five days after application was similar to that of the controls. Similar results were shown in a study by Alves et al. (2002), where *M. anisopliae *caused no mortality in *Cx. quinquefasciatus *Say larvae introduced four days after the spores were applied [13]. This is in contrast to Pereira et al. (2009), who found *M. anisopliae *spores to cause 50% mortality in *Ae. aegypti *larvae exposed to fungal spores that were applied ten days previously [34]. The studies mentioned here were carried out in controlled climate conditions (25-27°C) in the laboratory. In field conditions the spores are more likely to lose their pathogenicity in less time due to exposure to hight temperatures and UV-radiations. This may explain why unformulated fungal spores did not cause any significant reduction in pupation in the field bioassays, where the water surface temperatures were measured to be as high as 38.8°C. The measured (water surface) temperatures agree with those reported by Paaijmans et al. (2008) for similar sized water-bodies and are known to exhibit high daily fluctuations [44].

When the larvae were exposed to fungal spores on the same day as the spores were applied, unformulated spores and spores formulated in WaterSavr or Shellsol T caused larval mortality over the next few days. However, only fungal spores formulated in ShellSol T caused significantly higher mortality in larvae introduced seven days after the fungal spores had been applied. Fungal spores formulated in ShellSol T remained pathogenic possibly because ShellSol T prevented spores from absorbing the amount of moisture required to stimulate germination [21,31]. ShellSol T was also considered a good carrier of fungal spores in other studies [31,45]. WaterSavr, on the other hand, did not protect fungal spores.

ShellSol T was the only formulation that we tested in the field as the laboratory results showed high persistence of pathogenicity in the fungal spores formulated only with this product. Unformulated *M. anisopliae *and *B. bassiana *did not suppress the larval population effectively in the field. In contrast to the situation in the laboratory, the spores were exposed to sunlight, rain and fluctuating temperatures in the field which might have reduced spore survival. By contrast, only 10-20% of the larvae treated with spores formulated in ShellSol T, developed into pupae. Both *M. anisopliae *and *B. bassiana *spores were found to be equally effective when formulated in ShellSol T. Oil formulations are known to improve spore survival, improve fungal efficacy against insects and reduce spore sensitivity to UV radiation [31,45].

In the field residual effect of formulated spores could not be tested after a certain number of days because the plastic containers began to harbour *Culex *larvae and thus had to be drained. The presence of *Culex *larvae is an indication that ovipositing female *Culex *mosquitoes were not repelled by the fungus treatment. It is disadvantageous for a larval control agent to have an oviposition-repellent effect because in that case ovipositing mosquito females are forced to seek and deposit their eggs at alternative untreated sites. This means that the control agent only targets the existing larval population and needs to be reapplied after the site has been inhabited again. Studies specifically designed to establish the response of ovipositing anopheline female mosquitoes to fungal spores and the residual effect of fungal spore treatment are required for a better understanding. Oil-formulated *M. anisopliae *spores have been shown to have an increased ovicidal activity in case of *Ae. aegypti *eggs [46]. This might be an added advantage if anopheline eggs are also affected by *M. anisopliae *spores similar to the *Ae. aegypti *eggs.

Pathogenicity of control agents in the field is generally lower than that in the laboratory settings [47]. In the field bioassays, therefore, a higher dose (20 mg/450 cm^2^) of fungal spores was also tested together with the dose tested in the laboratory (10 mg/441 cm^2^). The laboratory dose, however, showed similar efficacy in the field by reducing pupation similar to the higher dose. Therefore doses lower than used in the current study should be evaluated to establish the lowest effective amount of fungal spores required to treat a certain area.

ShellSol T was a candidate carrier that not only facilitated the application of spores but also improved their efficacy by providing maximum chance for contact (spreading the spores on the water surface) with the larvae and increasing spore persistence. The fungal spores readily suspend in ShellSol T with a slight agitation. This is advantageous as the spores can be conveniently mixed in ShellSol T, on the spot, which means that during transport and storage only the bio-active agent would have to be kept at low temperatures rather than the whole mixture. This can reduce the cooling space requirement as ShellSol T itself is a stable product and has no particular storage demands. It has been shown that the percentage germination of dry spores is generally higher than that of oil-formulated spores when stored at the same temperature for the same number of days [[23]; unpublished data]. The fungal spores *Metarhizium flavoviride *had a germination rate of 80% when stored at 30°C for 90 days as compared to 90% when stored dry under similar environmental conditions [23]. In this context, it seems more efficient to store fungal spores separately and only mix them with the oil-component shortly before application.

The results of this study show the necessity of a good formulation for fungal spores when these are to be utilised in the field. The efficacy of unformulated (dry) spores was so low in the field situation that their application, as such, is not justified. While ShellSol T-formulated spores were highly effective in killing anopheline larvae in the field an important point to consider is the potential increased risk to the non-target organisms due to their improved persistence and/or undesirable properties of the solvent [33,48-50]. ShellSol T has a low toxicity effect on fish, aquatic invertebrates and microorganisms at concentration higher than 1 g/liter [51]. Considering the volume of ShellSol T that we tested (200-230 μl on 1 L of water), the concentration of ShellSol T was 0.15 g/L which is nearly seven times lower than the lowest lethal concentration. ShellSol T evaporates and therefore is less likely to remain in the aquatic habitats. Detailed safety studies, however, are necessary to have a better understanding of any adverse effect ShellSol T might have on the environment and non-target organisms, at the required doses.

Besides formulation, it is very important to identify the best delivery method (where, when and how) to fully utilize the entomopathogenic potential of *M. anisopliae *and *B. bassiana *spores. Frequency of re-application has to be determined based on the residual effect of formulated spores in the field. The feasibility of applying formulated spores at artificial breeding sites, baited to attract ovipositing females, is also worth testing [52]. A good delivery system will reduce the chances of non-target organisms coming into contact with fungal spores.

## Conclusions

From a number of candidate products tested for the formulation of entomopathogenic fungi, ShellSol T emerged as a promising carrier of fungal spores when targeting anopheline larvae. Spores of *B. bassiana *and *M. anisopliae *formulated in ShellSol T had an increased efficacy against larvae of *An. gambiae s.s*. as compared to unformulated spores and were also more persistent under field conditions in Kenya. Other oils with physical properties similar to ShellSol T may also serve as good carriers. Together with a sound delivery system, these formulated fungi can be utilised in the field, providing additional tools for biological control of malaria vectors.

## Competing interests

The authors declare that they have no competing interests.

## Authors' contributions

TB designed the study, carried out the experimental work, performed the statistical analysis and drafted the manuscript. CJMK helped with the study design, statistical analyses, and drafting the manuscript. WT provided scientific guidance in interpretation of the findings and drafting the manuscript. All authors read and approved the final manuscript.
